# Metastatic rectal cancer to papillary thyroid carcinoma: a case report and review of literature

**DOI:** 10.1186/s12876-020-01286-z

**Published:** 2020-05-06

**Authors:** Min Luo, Yu Huang, Yongqiang Li, Yumei Zhang

**Affiliations:** Department of Medical Oncology, Guangxi Medical University Cancer Hospital, No.71, Hedi road, Nanning, 530021 PR China

**Keywords:** Rectal cancer, Colorectal cancer, Papillary thyroid carcinoma, Secondary thyroid neoplasm, Tumor-to-tumor metastasis

## Abstract

**Background:**

Tumor-to-tumor metastasis is a rare event. Rectal cancer to primary thyroid neoplasm metastasis is extremely rare. Herein, we reported a case of metastatic rectal adenocarcinoma to a papillary thyroid carcinoma. The incidence and clinicopathological characteristics of metastatic colorectal cancer to a thyroid gland neoplasm were described, and the pertinent literature was reviewed.

**Case presentation:**

A 34-year-old female patient had curative treatment of initial rectal adenocarcinoma in 2012, and was found to have lung metastases by follow-up CT scan 3 years later. In 2018, she was found to have thyroid metastasis by imaging due to left neck pain and hoarseness. A fine-needle aspiration biopsy (FNAB) result suggested suspicious papillary thyroid carcinoma (PTC). The patient underwent a total thyroidectomy and bilateral cervical lymph nodes dissection. The histopathology of thyroidectomy specimen revealed a rectal adenocarcinoma metastatic to the thyroid concomitant with the papillary carcinoma in metastatic adenocarcinoma. The patient received levothyroxine supplementation therapy and palliative chemotherapy with irinotecan and anti-angiogenesis for the metastatic rectal adenocarcinoma. After 1 year of thyroidectomy, no newly developed lesion evidence of recurrent PTC was observed. The patient remains still alive.

**Conclusion:**

The possibility of metastases should be considered in patients with a history of rectal cancer and with a thyroid lesion, particularly in those with ageing, hereditary nonpolyposis colorectal cancer (HNPCC) or long-term survival. The diagnosis should be histologically confirmed for the presence of both primary thyroid lesions and secondary thyroid neoplasms. Thyroidectomy may be a feasible treatment for symptomatic thyroid metastasis or thyroid cancer. we need to gain more available evidence from large or multi-center clinical data to help clinicians to diagnose rectal cancer to thyroid neoplasm metastases and evaluate treatment.

## Background

Colorectal cancer (CRC), with an estimated number of over 1.8 million new cases and 881,000 cancer-related deaths worldwide in 2018, ranks the third most commonly diagnosed cancer but is the second leading cause of cancer-related death in the world [1]. Notably, 21–26% of CRC patients in the United States had distant metastasis at diagnosis and the overall survival rate in patients with metastatic colorectal cancer (mCRC) was 12% at 5 years [2]. The common sites of rectal cancer include regional lymph nodes, the liver, and the lungs, but metastasis to the thyroid gland is rare. And furthermore, rectal cancer to primary thyroid neoplasm metastasis, a tumor-to-tumor metastasis, is extremely rare. Up to date, only eight documented cases of mCRC to primary thyroid neoplasm metastasis were reported [3–10]. We report here a case of metastatic rectal adenocarcinoma to the papillary thyroid carcinoma and review the related literature on tumor-to-tumor metastasis of CRC in the thyroid gland.

## Case presentation

**Fig. 1:**
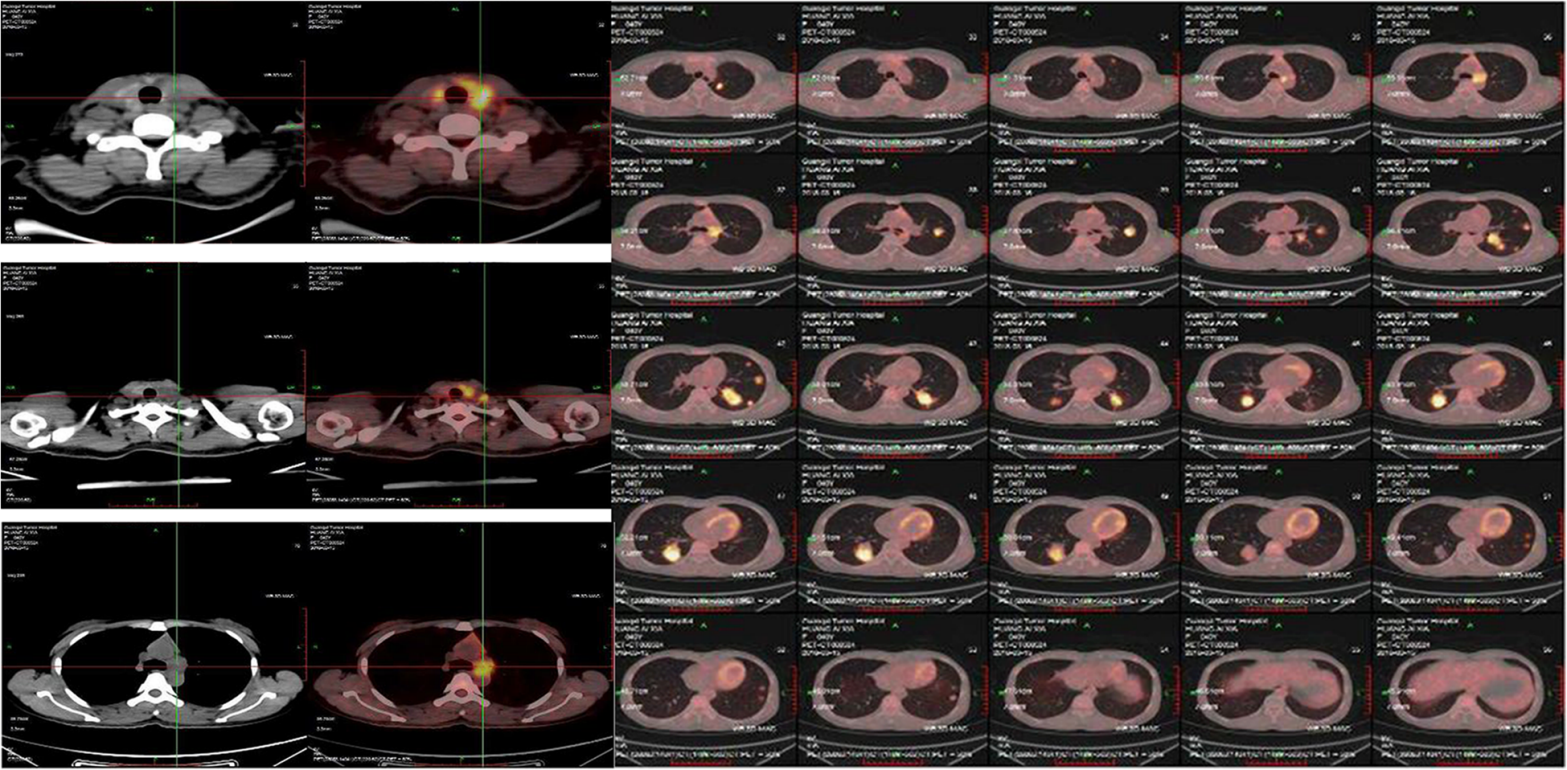
The ^18^F-FDG PET/CT scan images showing increased focal FDG uptake in the multiple thyroid masses, cervical lymph nodes, the fifth region mediastinal lymph nodes and multiple pulmonary nodules

**Fig. 2:**
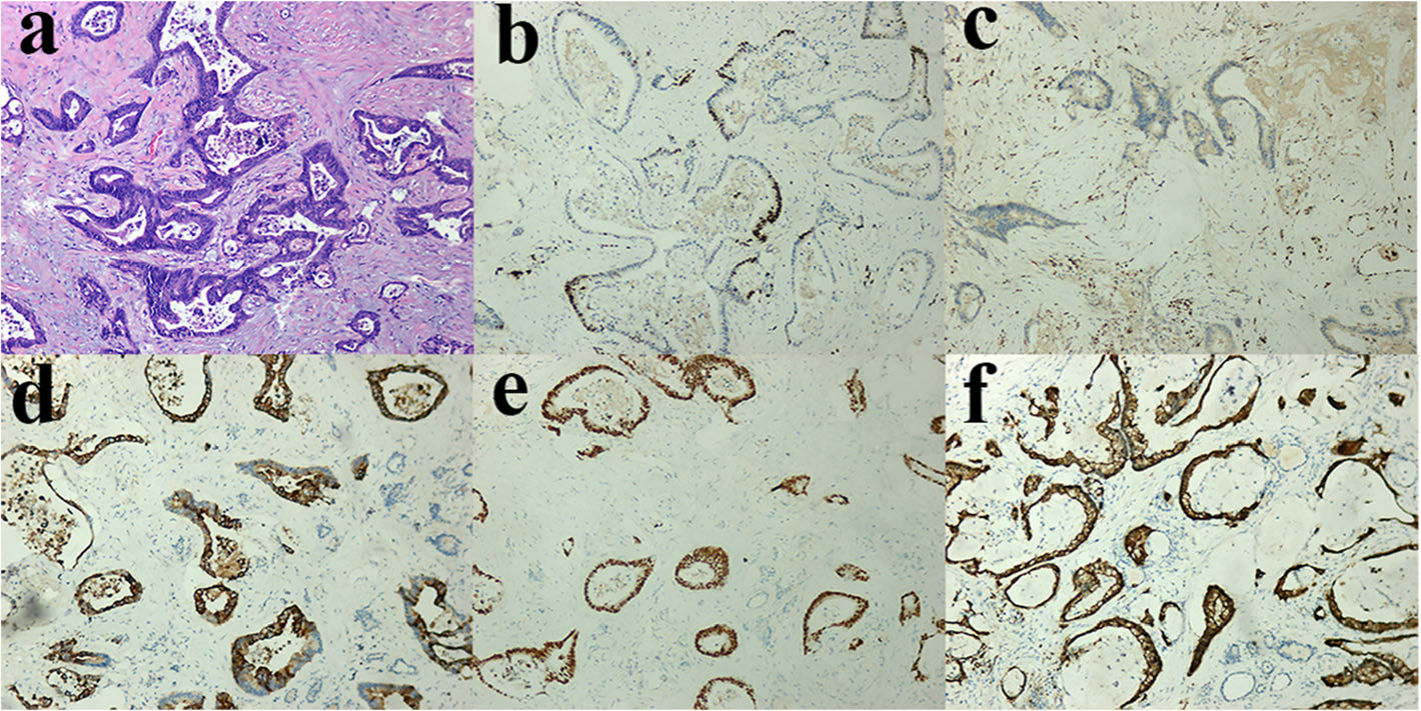
Images of histopathological and immunohistochemical (IHC) showing metastatic carcinoma in the resected thyroid gland. **a** H&E stain of thyroid gland detects adenocarcinoma with mucinous features, consistent with metastatic rectal adenocarcinoma. **b** Tumor cells are negative for TTF-1 staining. **c** Tumor cells are negative for PAX8 staining. **d** Tumor cells are positive for CK20 staining. **e** Tumor cells are positive for CDX-2 staining. **f** Tumor cells are positive for Villin staining.

**Fig. 3:**
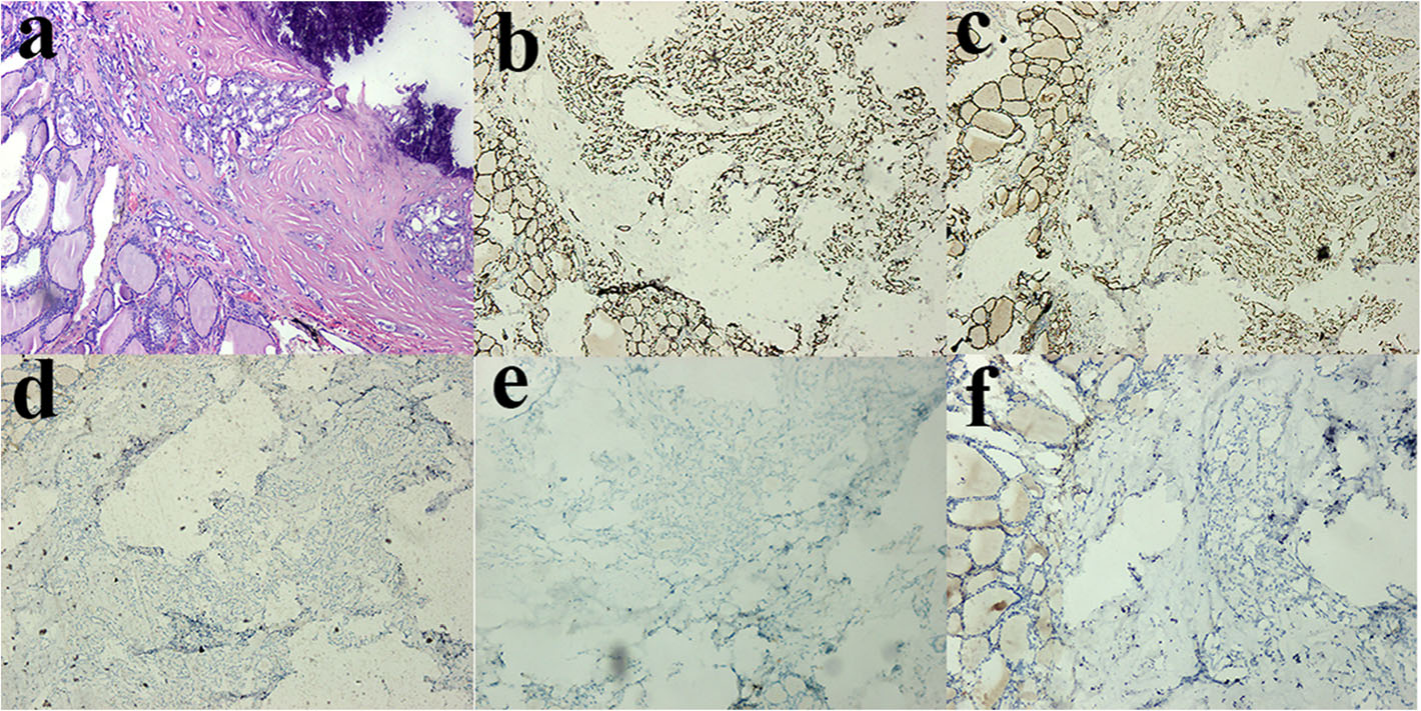
Images of histopathological and immunohistochemical (IHC) showing primary papillary carcinoma in metastatic adenocarcinoma. **a** H&E stain of primary papillary thyroid carcinoma. **b** Tumor cells are positive for TTF-1 staining. **c** Tumor cells are positive for PAX8 staining. **d** Tumor cells are negative for CK20 staining. **e**Tumor cells are negative for CDX-2 staining. **f** Tumor cells are negative for Villin staining

A 34-year-old female patient had an abdominal perineal radical resection (Miles operation) of a rectal cancer in February 2012, with a normal carcinoembryonic antigen (CEA) level of 3 ng/mL (reference range: <5.2 ng/mL). The postoperative pathology revealed a moderately differentiated rectal adenocarcinoma, with obvious invasion to posterior vaginal wall and regional lymph node metastases (pT4bN1M0, stage IIIC, Dukes C). The patient had adjuvant chemotherapy with CapeOx (capecitabine and Oxaliplatin) for six cycles postoperatively followed by chemoradiation. No evidence of tumor recurrence and metastasis was detected by fluorine-18-fluorodeoxyglucose-positron emission tomography integrated with computed tomography (^18^F-FDG PET/CT) scan in January 2013. Her disease recurred in February 2015 when bilateral pulmonary metastasis was found by follow-up CT scan. Intermittent oral chemotherapy of capecitabine was performed at the patient’s request from March 2015. In June 2017, she noted gradual enlargement of the anterior cervical bump, until she presented with left neck pain and hoarseness (in February 2018). Physical examination revealed a hard and diffuse goiter, which had a size of 3 × 3 cm, moved with deglutition. At this time, blood tests revealed an elevated CEA (19 ng/ml) and normal thyroid function. The neck ultrasound showed multiple heterogeneous hypoechoic nodules in the thyroid gland, particularly at the expense of the left lobe measuring 23 × 17 mm, and swollen bilateral cervical lymph nodes. A ^18^F-FDG PET/CT scan revealed increased focal FDG uptake in the multiple thyroid masses (maximum standard uptake value [SUV max] 9.7), multiple cervical lymph nodes (SUV max 7.8), enlarged lymph nodes in the fifth region of mediastinum (SUV max 7.3), and multiple nodules in both sides of the lung (SUV max 12.6) (Fig. 1). There was no evidence of liver metastasis or abdominal masses. A fine-needle aspiration biopsy (FNAB) showed suspicious papillary thyroid carcinoma (PTC). The patient then underwent a total thyroidectomy and bilateral cervical lymph nodes dissection. Intraoperatively, the tumor was found to involve the left recurrent laryngeal nerve. The histopathology of thyroidectomy specimen revealed moderately differentiated adenocarcinomas in the left and right thyroid lobes, which had the same histology as the primary rectal adenocarcinoma. In the right thyroid lobe, an incidental concomitant presence of primary papillary carcinoma (diameter 5 mm) was detected in metastatic adenocarcinoma. Immunohistochemical (IHC) staining was performed using thyroid transcription factor-1 (TTF-1), paired box protein 8 (PAX8), cytokeratin 20 (CK20), caudal-related homeobox transcription factor-2 (CDX-2) and villi protein (Villin) in order to clarify the origin of the tumor. The tumor cells of metastatic rectal adenocarcinoma were positive for gastrointestinal markers CDX2, CK20 and Villin but were negative for TTF-1 and PAX8 (Fig. 2). In contrast, the tumor cells in the PTC were positive for TTF-1 and PAX8, but negative for rectal adenocarcinoma marker (Fig. 3). Histopathological and IHC examinations of the neck lymph nodes were consistent with the diagnosis of rectal adenocarcinoma metastasis. The histological diagnosis of this patient was papillary thyroid carcinoma pT1aN0M0, Stage I, and multiple metastatic rectal adenocarcinoma rT4bN1M1b, stage IVB. Due to metastatic rectal adenocarcinoma, RAS, *BRAF* V600E and PI3K molecular assay was performed and revealed that NRAS, *BRAF* V600E and PI3K was wild type but KRAS exons 2 had a mutation. Therefore, the patient was not eligible for cetuximab treatment, which was the inhibitor of the epidermal growth factor receptor (EGFR). She received levothyroxine supplementation therapy and palliative chemotherapy with irinotecan and anti-angiogenesis for the metastatic rectal adenocarcinoma.

After 1 year of thyroidectomy, no evidence showed newly developed lesion of recurrent PTC. Currently, the patient remains alive and receives Regorafenib to prevent the aggravation of pulmonary metastases.

## Discussion and conclusions

Due to the fast flow of arterial blood and the high iodine and oxygen content in the thyroid gland tissue, metastasis of a cancer to the thyroid gland is a rare event in clinical practice, which comprises approximately 1.3–3% of all thyroid malignant neoplasms [11, 12]. However, autopsy series reported the overall incidence of the secondary thyroid malignancies as high as 24% in the populations with known primary cancers [13]. While metastasis of CRC to the thyroid gland is uncommon, which only accounts for about 0.1% of CRC cases [14], metastasis to an intrathyroidal primary neoplasm (i.e. tumor-to-tumor metastasis), is exceptionally uncommon. Tumor-to-tumor metastasis is defined as the metastasis of one tumor into another, including both malignant-to-benign tumor metastasis and malignant-to-malignant metastasis. The first case of tumor-to-tumor metastasis was first reported by Berent in 1902 [15]. To diagnose a tumor-to-tumor metastasis requires the following criteria as [16–19]: 1) more than one primary tumor must exist; 2) the recipient tumor must be a true benign or malignant neoplasm; 3) the donor malignancy must be a true metastasis; and 4) the exception of contiguous growth of one tumor into another adjacent tumor (“collision tumor”), embolization of tumor cells, and metastasis to the lymphatic system that were already involved by generalized lymphatic or hematological malignancy. In the present report, our patient appeared to have satisfied all these criteria based on the second pathologic examination of PTC, which was presented 6 years after curative treatment of the initial rectal adenocarcinoma, and the patient had a history of visceral metastases.

To the best of our knowledge, fewer than 50 case reports of tumor-to-tumor metastasis in which the recipient tumor was a primary thyroid neoplasm were reported in the literature. Furthermore, only eight documented cases were CRC to primary thyroid neoplasm metastasis (Table 1). Our present report is the ninth case to describe mCRC metastasis to thyroid with a PTC. The first documented case of mCRC to a primary thyroid neoplasm was described by Kameyama in 2000 [3]. Of the nine cases of mCRC to the recipient thyroid neoplasm reported, 2 the recipient thyroid tumors were follicular adenomas [3, 5], 1 was Hürthle cell carcinoma [4], 4 were papillary carcinomas [6, 7, 9], 1 was medullary carcinoma [8], and 1 was follicular carcinoma [10]. Our literature review found that the interval between diagnosis of primary CRC and its metastasis to a thyroid neoplasm was in a range of 18 months to 9 years, mainly after curative treatment of the first diagnosis; only 1 case was detected synchronously with the primary malignancy, metastasis to a thyroid follicular adenoma as the initial presentation of a colonic adenocarcinoma [5]. Among these cases, thyroid metastasis was usually present with other sites distant metastases, especially lung metastases. Keranmu et al. reported that 81.0% of patients with mCRC to thyroid showed concomitant lung metastasis [20]. In our case, lung metastases appeared to be the first sign of hematogenous spread after 3 years from the primary diagnosis. Therefore, hematogenous dissemination can be considered to be the main pathway for rectal cancer to the thyroid gland. The “seed and soil” theory suggests that tumor or metastasis develop when viable tumor cells (the “seed”) can proliferate at the favorable growth environment of specific organs or the recipient tumors (the “soil”) via hematogenous dissemination [3, 15]. Because of rich vascularity of thyroid gland, thyroid neoplasms are the common recipient tumors in tumor-to-tumor metastasis. The literature review also found two cases had isolated CRC to a thyroid neoplasm metastasis with a history of thyroid goiter [4, 7]. Based on the Batson’s new theory of vertebral venous metastasis [21], we hypothesized that tumor cells migrate through the vertebral veins and directly enter to the thyroid without entering the thoracic and abdominal cavity, or without involving the caval vein, pulmonary vein and portal vein. In fact, the thyroid diseases are vulnerable to primary or secondary thyroid cancer growth due to cancer growth-induced decreases in blood flow and levels of oxygen and iodine, such as Hashimoto’s thyroiditis. However, in the reported patients with mCRC to intra-thyroidal thyroid neoplasm metastasis, we found that the majority of patients had normal thyroid gland function. Perhaps, multinodular goiters and adenomas that change the structure, could also alter the thyroid environment so that it becomes a favorable site for metastatic colorectal cancer cells to settle and grow.

**Table 1:** Reported cases of colorectal cancer metastasis to thyroid neoplasm in the literature

Author and publish year	Age (year)/gender	Primary site	History with thyroid	Clinical symptoms of thyroid tumor	FNA diagnosis	Thyroid tumor	CEA level (ng/ml)	Interval from primary cancer diagnosis	Metastatic Organs	Treatment of thyroid tumor	Prognosis after thyroid tumor diagnosis
Kameyama 2000 [3]	82/M	sigmoid colon	NA	Autopsy	NA	Microfollicular adenoma	217	2 years	Multiple organs	None	Dead
Witt 2003 [4]	71/M	colon	a long-standing thyroid goite	Thyroid mass, airway obstruction	Benign thyroid goiter	Hürtle cell carcinoma	NA	7 years	Thyroid	Thyroidectomy	NA
Fadare 2005 [5]	59/F	sigmoid colon	a thyroid nodule 23 years	Enlarged left thyroid lobe	Follicular neoplasm	Follicular adenoma	NA	Synchronous	Liver and thyroid	Subtotal left thyroidectomy	>2 years
Cherk 2008 [6]	52/M	rectal	NA	Asymptomatic+ PET/CT	PTC	PTC	9.7	18 months	Lung and thyroid	Right hemi-thyroidectomy	NA
Jin 2014 [7]	62/F	rectal	goiter over 50 years	Goiter, dyspnea	NA	PTC	22.68	21 months	Thyroid	Partial thyroidectomy	>2 years
Yeo 2014 [8]	53/M	sigmoid colon	NA	Asymptomatic + PET/CT + ultrasound	MTC	MTC	17.3	2 years	Lung and thyroid	Total thyroidectomy and bilateral central neck dissection	1 year
Amenduni 2014 [9]	63/F	left side colon	NA	Cervical swelling, dyspnoea	NA	Papillary microcarcinoma	NA	2 years	Thyroid	Total thyroidectomy	NA
Melis 2018 [10]	53/M	colon	NA	Asymptomatic+ MRI	Follicular neoplasm + mCRC	Follicular carcinoma	NA	9 years	Thyroid	Thyroidectomy	>1 year
Current case 2019	34/F	rectal	NA	Cervical bump, neck pain and hoarseness	PTC	PTC	19	6 years	Lung and thyroid	Total thyroidectomy and bilateral cervical dissection	>1 year

Note: *NA* Not available or not clearly stated, *MTC* Medullary thyroid carcinoma, *PTC* Papillary thyroid carcinoma, *mCRC* Metastasis colorectal cancer

Thyroid metastases from rectal cancer may clinically present as an enlarged thyroid gland, neck mass, dysphagia, dry cough, dyspnea and hoarseness, which are similar to those of the primary thyroid malignancy. Through the literature review, we found that 5 of the all cases reported had these clinical features, and that another 4 cases were discovered as incidental findings with elevated CEA levels or during follow-up by accurate imaging examinations. CEA is one of the most widely used tumor markers, and its increase in the serum may be used for disease monitoring and evaluation of efficacy, especially for postoperative monitoring of CRC [22]. Elevated CEA levels >5 ng/mL are helpful in detecting early recurrences and advanced stage in patients with resected rectal cancer [23, 24]. The National Comprehensive Cancer Network (NCCN) guidelines recommended that PET/CT scan should be more commonly used in detection of recurrent rectal cancer in patients with elevated CEA. A recent systematic review and meta-analysis of 11 studies (510 patients) found that PET/CT can be detect tumor recurrence with a sensitivity of 94.1%and a specificity of 77.2% [25]. With widely clinical use of PET/CT scan, the incidence of newly detected thyroid lesions is increasing. Previous reports described that the incidence of thyroid incidentaloma identified by PET/CT was 1.2 to 3.1% in non-thyroid disease patients and 14 to 59.8% of them were proven to be malignant, with papillary carcinomas being the common type by histopathology [26–28]. Although the accuracy of thyroid imaging has improved with the introduction of PET/CT scan, the accurate diagnosis required histology to differentiate between primary thyroid lesions and secondary thyroid neoplasms.

Presently, FNAB is the front-line method in clinic to evaluate a suspicious thyroid mass. Cytological evaluation from FNAB usually provides a rapid and reliable diagnosis of primary thyroid malignancy due to favorable sensitivity (83%) and specificity (92%), which is the same as metastatic thyroid neoplasm due to high accuracy (87%) [29, 30]. In 15–30% cases, FNAB had difficulty with diagnosis, due to the issues with distinguishing the primary tumors from poorly differentiated metastatic thyroid tumors such as anaplastic thyroid carcinoma. Witt RL.et al. [4] reported a case in which FNAB diagnosed a benign thyroid goiter, but final pathologic examination indicated a colon adenocarcinoma metastatic to a thyroid Hürthle cell carcinoma with focal areas of dedifferentiated anaplastic thyroid cancer. In situations like this, IHC and molecular analysis may be useful for improving the accurate distinction between a metastatic and a primary tumor. The current study demonstrated the importance of IHC for confirming tumor-to-tumor metastasis, which showed that CDX2, CK20 and Villin were positive in metastatic rectal adenocarcinoma, and that TTF-1 and PAX8 were positive in PTC. Immunostaining for the thyroid specific biomarkers TTF-1, thyroglobulin or PAX8 can be used to differentiate between primary and secondary thyroid neoplasm. PAX 8 is an important transcription factor for thyroid organogenesis and a useful immunohistochemical marker for thyroid tumors; it is also a well-recognized marker of Müllerian tract and kidney tumors. PAX 8 can assist in distinguishing thyroid carcinomas from lung adenocarcinomas, and its sensitivity for differentiated thyroid tumors is similar to that of thyroglobulin and TTF-1 [31]. Also, PAX 8 has been reported to be positive in 74% of anaplastic thyroid carcinomas, whereas these tumors rarely express thyroglobulin and TTF-1 [32, 33]. Rectal cancer can be CK20 and CDX-2 positive, which are useful to confirm the gastrointestinal etiology, but is thyroglobulin and calcitonin negative. As in the present case, while FNAB cytology made suspicious diagnosis, histological examination of thyroidectomy confirmed it, which was further supported by specific immunohistochemical markers. Thus, specific immunohistochemical markers can be very useful in the correct diagnosis of rectal cancer to thyroid carcinomas.

Thyroidectomy is a conventional treatment for primary thyroid malignancy, is safe and may improve survival. However, thyroid metastasis appears to be an advanced disease stage, and surgery may only serve as palliative treatment. Currently, how to appropriately manage patients with thyroid metastasis remains controversial. Romero Arenas et al. [34] reported that the median overall survival (OS) was significantly longer in patients with thyroid metastasis who underwent thyroidectomy than in the nonoperative patients (34 vs. 11 months), indicating that thyroidectomy may still be beneficial for patients with thyroid metastasis. It is highly likely that the therapeutic effects of surgical management may depend on a number of factors, including the site, the stage and the performance of the primary tumor, and the symptoms caused by the thyroid mass. Of the 8 cases reported in the literature, the patients had either total or subtotal thyroidectomy. All of these patients had relieved symptoms and no one had recurrence. Russell et al. [35] recently showed that locoregional recurrence was more likely in patients with thyroid metastasis treated with subtotal thyroidectomies (13.3%) versus total thyroidectomies (4.8%). Even if thyroidectomy may be a palliative procedure, it can be beneficial to quality of life, reduce the incidence of locoregional recurrence and improve long-term survival. Although distant metastases are often an adverse prognosticator, thyroid metastasis may not have poor prognosis. Overall prognosis appears to be most closely associated with the advanced primary tumor. In the present case, although our patient had recurrent laryngeal nerve palsy and pulmonary metastasis of rectal adenocarcinoma, surgery relieved her clinic symptoms and controlled the local recurrence. Thus, in our case, the prognosis of this patient appeared to be determined by the patient’s metastatic rectal adenocarcinoma rather than the primary thyroid malignancy.

Through the literature review, we found that, an average age of patients with CRC to thyroid tumor metastasis was 59.2 years (ranging from 34 to 85) and that CRC to thyroid tumor metastasis did not seem to have gender predilection. This observation was not consistent with that reported by Froylich et al. [36], who found that two thirds of the patients were female in the 34 cases of metachronous colon metastasis to the thyroid, indicating that a slight female gender predisposition might exist. In the series study by Papi et al. [12], the majority of the secondary lesions of the thyroid were diagnosed at a median age of 66 years, which was correlated to the increased risk of developing a malignant tumor with ageing. As reported by Kondo et al. [37], the risk of developing new primary cancer in cancer survivors, depending on age, was increased at least by 20%. Lynch syndrome (also known as hereditary nonpolyposis colorectal cancer [HNPCC]) is an autosomal dominant condition that leads to increased risks for colorectal cancer, accounting for 2 to 5% of all colon and rectal cancers [38–41]. This hereditary syndrome results from a germline mutation in one of the DNA mismatch repair (MMR) genes (MLH1, MSH2, MSH6, and PMS2). Pelizzo et al. [42], revealed that both colonic and thyroid cancers were more likely to occur in association with MLH1 or MSH2 germ-line mutations in HNPCC patients. Therefore, additional thyroid monitoring should be performed in the post-treatment surveillance of patients with rectal cancer, particularly in those with ageing, HPNCC or long-term survival.

Metastatic rectal carcinoma to a primary thyroid malignancy is exceptionally uncommon. The frequency of rectal cancer to thyroid neoplasm metastasis is increasing due to the improved diagnostic technologies and long-term survival. The possibility of rectal cancer to thyroid neoplasm metastases should be considered in patients with a history of rectal cancer and with a thyroid lesion. Histological examination of thyroid specimen, histomorphology in comparison with the prior primary rectal cancer, specific immunohistochemical and molecular markers are the keys to diagnose rectal cancer to thyroid tumor metastasis. Thyroidectomy may be a feasible treatment for symptomatic thyroid metastasis or thyroid cancer which would relieve the clinical symptoms. However, these recommendations may not be translatable into clinical, for which further work is required to develop a standardized, reproducible and valid evaluation methodology. we need to gain more available evidence from large or multi-center clinical data, such as retrospective cancer center records or the National Cancer Institute’s Surveillance Epidemiology and End Results (SEER). It is hoped this proposed approach will help clinicians to diagnose rectal cancer to thyroid neoplasm metastases and evaluate treatment.

## Data Availability

N/A

## Abbreviations

CRC: Colorectal cancer
mCRC: Metastatic colorectal cancer
CEA: Carcinoembryonic antigen
18F-FDG PET/CT: Fluorine-18-fluorodeoxyglucose-positron emission tomography integrated with computed tomography
SUV max: Maximum standard uptake value
FNAB: Fine-needle aspiration biopsy
PTC: Papillary thyroid carcinoma
IHC: Immunohistochemical
TTF-1: Thyroid transcription factor-1
PAX8: Paired box protein 8
CK20: Cytokeratin 20
CDX-2: Caudal-related homeobox transcription factor-2
Villin: Villi protein
EGFR: Epidermal growth factor receptor
NCCN: National Comprehensive Cancer Network
OS: Overall survival
HNPCC: Hereditary nonpolyposis colorectal cancer
MMR: DNA mismatch repair
